# Supplementary material for: TCF19 Impacts a Network of Inflammatory and DNA Damage Response Genes in the Pancreatic β-Cell
Source: Metabolites. 2021 Aug 4;11(8):513. doi: 10.3390/metabo11080513 (PMC8400192; doi:10.3390/metabo11080513)
Supplement: Supplementary file 1 [file metabolites-11-00513-s001.zip › Supplementary Table S2.pdf]

Supplementary Table S2: Primer sequences used for real-time PCR experiments

Gene	5' Primer	3' Primer
**Human parp9**	GGCAGACGGCAGATGTAATTG	GAAACTGTTTAGCCTTTGTGGCAAG
**Human dtx3l**	TCAGTGAAAGGGCAGCTAAGG	GAAATTTGAGGTCTCGTGTTCTTC
**Human ddx60**	CTTCAAGGATGCCGAGTATGCGT	CTTCCAAGAAACTTCCCCACTCT
**Human mx1**	GGAAATTAATAAAGCCCAGAATG	AAGATTCCGATGGTCCTGTC
**Human usp18**	TGTGCACTTGGTGGAGAGAC	GAAAGTGGGAGGGTGAGCAT
**Human Tcf19**	AGAAACTCCGTGTAGACAAAGCCC	ATGGAACCAGACGTCACAGCCAT
**Rat ccnA1**	AACCACCAACCAGTTCCTCCTTCA	TCCAGAAGGCTCAGTTCTGCAACA
**Rat ccnA2**	ATGAAGAGGCAGCCAGACATCACT	ACAGCCAAATGCAGGGTCTCATTC
**Rat ccnB1**	TGTGTCAGGCTTTCTCCGATGTGA	TTTCCAGTGACTTCACGACCCAGT
**Rat ccnD1**	TGCTGCAAATGGAACTGCTTCTGG	AAGGTCTGTGCATGTTTGCGGATG
**Rat ccnD2**	ACTTCAAGTTTGCCATGTACCGCG	TTAAGCAGCACAGCCTCGATTTGC
**Rat ccnD3**	TCACTGCATTTGGATCTGGGTCCT	ACCCTCAACCACAGAAGCATAGCA
**Rat ccnE1**	TTCCCATGGAAGACTCCCACAACA	ATGGCAGGTCTGGTCATTCTGTCT
**Rat ccnE2**	AACCAGCCAGACTCTCCACAAGAA	TGCAAGGACTGATTCCTCCAACA
**Rat FoxM1**	AGCTCTTCCAAGGCAAAGACAGGA	TTAACCCGATTCTGCTCCAGGTGA
**Rat cdk4**	GTTGCTGCTGGAAATGCTGACCTT	GCTGCCACTTCAGCAAGGTTCTTT
**Rat Plk1**	TGCAGTACATAGAGCGTGATGGCA	TGTGCGGAACCATGTTCGTAGGTA
**Rat Ki67**	AAGAACCCACACAGATGCCCTGTA	TCGCACTTTGCCTTGATGTTGG
**Rat cdkn2c**	TGCGCTGCAGGTTATGAAACTTGG	GGCAGCATCGTAAATGACAGCAAA
**Rat cdkn1a**	GCGGGACCGGGACATC	CGCTTGGAGTGATAGAAATCTGTTAG
**Rat cdkn1b**	GCCTTCAATTGGGTCTCAGGCAAA	AAGAATCTTCTGCCGCAGGTCG
**Human and rat beta actin**	TCAAGATCATTGCTCCTGAGC	TTGCTGATCCACATCTGCTGGAAG
